# Two-Dimensional Permutation Vectors’ (PV) Code for Optical Code Division Multiple Access Systems

**DOI:** 10.3390/e22050576

**Published:** 2020-05-20

**Authors:** Hassan Yousif Ahmed, Medien Zeghid, Waqas A.Imtiaz, Teena Sharma, Abdellah Chehri, Paul Fortier

**Affiliations:** 1 Electrical Engineering Department, College of Engineering at Wadi Aldwaseer, Prince Sattam Bin Abdulaziz University, Al-Kharj 16278, Saudi Arabia; hassanuofg@gmail.com (H.Y.A.); m.zeghid@psau.edu.sa (M.Z.); 2 Electronics and Micro-Electronics Laboratory (E. μ. E. L), Faculty of Sciences, University of Monastir, Monastir 5000, Tunisia; 3 Department of Electrical Engineering, Jalozai Campus, University of Engineering and Technology, Peshawar 23431, Pakistan; waqasahmed15@gmail.com; 4 Department of Applied Sciences, University of Quebec in Chicoutimi (UQAC), Chicoutimi, QC G7H 2B1, Canada; 5 Department of Electrical and Computer Engineering, Laval University, Québec, QC G1V 0A6, Canada; fortier@gel.ulaval.ca

**Keywords:** OCDMA, vector permutation, phase induced intensity noise, multiple access interference, cross-correlation

## Abstract

In this paper, we present a new algorithm to generate two-dimensional (2D) permutation vectors’ (PV) code for incoherent optical code division multiple access (OCDMA) system to suppress multiple access interference (MAI) and system complexity. The proposed code design approach is based on wavelength-hopping time-spreading (WHTS) technique for code generation. All possible combinations of PV code sets were attained by employing all permutations of the vectors with repetition of each vector weight (*W*) times. Further, 2D-PV code set was constructed by combining two code sequences of the 1D-PV code. The transmitter-receiver architecture of 2D-PV code-based WHTS OCDMA system is presented. Results indicated that the 2D-PV code provides increased cardinality by eliminating phase-induced intensity noise (PIIN) effects and multiple user data can be transmitted with minimum likelihood of interference. Simulation results validated the proposed system for an agreeable bit error rate (BER) of 10^−9^.

## 1. Introduction

Driven by the ever-growing data size from internet usage rate, optical code division multiple access (OCDMA) system became the center of attention due to its ability to overcome the bottleneck problem for data-hungry applications with massive data rate. OCDMA systems permit multiple subscribers to asynchronously and concurrently access the medium without any contention along with the ability to provide reliable bandwidth at relatively low cost [1,2].

Spectral amplitude coding (SAC) technique is measured as a proficient scheme in alleviating the effects of multiple access interference (MAI) and its inherited intensity noise [3]. Along with its MAI alleviation features, SAC-OCDMA system is implemented with low cost and less complexity with broadband incoherent sources such as light emitting diodes (LED) [4,5].

Two dimensional (2D) coding schemes are enthusiastically adapted to increase orthogonality which lead to efficient bandwidth utilization. Further, performance is improved in terms of increased subscribers due to spectral density expansion at the cost of high-speed electronic devices [6].

By combining two different dimensions of CDMA, two-dimensional (2D) codes have been proposed for OCDMA networks. SAC-based systems mostly use spectral/spatial (S-S) and wavelength/time (W-T)-coding approaches for 2D coding [7,8]. In the case of 2D (W-T) scheme, a different wavelength is assigned to each chip and placed across the bit time period, which further offers higher transmission capacity and more flexibility when compared with 1D coding schemes [9,10,11]. Further, to improve bit error rate (BER) and cardinality for a certain data rate, AND detector circuit is used at the receiver [12]. AND detector circuit composed of parallel structure of two photo detectors or photodiodes (PD and s-PD) which are connected electrically in opposition and power difference of the two optical inputs is the resultant output signal.

The 2D W-T optical orthogonal codes (OOC) with constant weight and variable code length properties were discussed by Yang et al. [13]. The 2D OOC code provides multimedia services with district service requirements for optical CDMA networks by designing encoder and decoders with fiber bragg gratings (FBGs).

A new family of 2D hybrid W-T code was proposed by Kandouci et al. [14], by combining OCC and balanced incomplete block design zero cross-correlation (BIBD-ZCC) codes. The 2D hybrid code provides both off-peak auto-correlation and cross-correlation features with time-spreading and wavelength-hopping patterns. The presented code is suitable for the asynchronous and synchronous OCDMA environment with benefits such as high cardinality and good correlation properties.

The 2D W-T OCDMA system, which is based on coherent light sources, was analyzed in the presence of beat noise (BN) and MAI effects by Bazan et al. [15]. For mitigating BN, a number of aspects for 2D Time/Wavelength (TW) code designs are presented along with BN dependence on code properties. Various 2D W-T codes based on single pulse per column/row and multiple pulse per column/row were compared. A new technique for designing OCDMA scheme with time spread and wavelength group hopping embedded maximum length (ML or m) sequence code was presented by Chang et al. [16]. This technique uses cyclic and periodic free-spectral-range properties of arrayed waveguide gratings (AWGs) routers to design 2D time-spreading and wavelength group-hopping embedded m-sequence code. The code showed cross-correlation ((λc) = G × w), where G stands for number of wavelengths and *w* referred as code weight. Fine AWG produces m-sequence code words, which is further time spread in the wavelength domain using a number of coarse AWGs.

Further, to design 2D zero cross-correlation (ZCC) code, W-T coding approach was used to obtain an increased number of subscribers by Kandauci et al. [17]. In this work, 2D ZCC codes were designed based on 1D ZCC codes while conserving same code length and correlation constraint. The proposed system architecture is simple and cost effective due to direct detection technique employed at the receiver.

Multi-diagonal prime hop code (2D MDPHC) based on W-T coding technique and with zero cross-correlation properties was presented by Panda et al. [18]. The presented code is a combination of two different 1D codes (MD and prime code) and can accommodate a larger number of users with an increased data transmission rate. The entropy in information theory explains the disorder of information. These types of imports are important and necessary because a well-developed identity in one discipline will bring definition to a phenomenon as well as mathematical tools and theory. Our aim here was to obtain crisp results in a simple setting which can be used to understand the basic trade-offs between the intrinsic entropy rate of the system and the available rate on the optical communication [19].

A passive optical network (PON) system based on 2D-OCDMA wavelength division multiplexing (WDM) was presented by Mrabet et al. [20] in which a hybrid of 2D prime hop system (PHS) codes and 2D hybrid coded (HC) signature codes were employed for uplink and WDM scheme for the downlink. A vertical cavity surface emitting laser was deployed with an avalanche photo detector receiver and optical line terminal unit in the optical network. The system performance was analyzed for the BER, signal to noise (SNR), optical power budget, and quality (Q) factor. BER improvement (10^−10^ at data rates 40 Giga bits per second (Gbps)) as well as long PON distance up to 41 km with increased maximum throughput equal to 285.1 Gb/s/km was achieved.

Moreover, Najjar et al. [21] proposed the construction of 2D diagonal eigenvalue unity (DEU) code using a spectral-spatial (S-S) coding approach with minimum cross-correlation. Results indicated that the 2D DEU provides an increase in cardinality when compared to existing 2D diluted perfect difference DPD and 1D DEU codes.

Mrabet et al. [22] presented an analytical model consisting of a hybrid of all-optical orthogonal frequency division multiplexing (AO-OFDM) and OCDMA. The presented model incorporated probabilistic subcarrier overlapping and MAI testing capabilities with over amplifier-free long-reach passive optical networks (LR-PONs) using cost-effective intensity modulations and direct detection (IM-DD) techniques. The proposed system considered subcarrier hopping by utilizing 2D-HC codes. It was observed that the hybrid (AO-OFDM) and OCDMA with 2D-HC outperformed a traditional multichannel OCDMA system for any number of simultaneous users and low received powers in comparison with 1D-Walsh Hadamard, 1D, and 2D prime codes. Numerical results showed that 16-quadrature amplitude modulation (16-QAM) AO-OFDM-OCDMA provides comparable performance to traditional multichannel 16-QAM coherent optical OFDM in the downstream direction. The presented technique supported up to 58 km with a maximum 45 users, without complex coherent technology. In addition, at 108 km as a maximum reachable distance (at 40 Gb/s), QAM signal was achieved in budget power calculation while considering standard forward error correction (FEC) techniques.

Further, a recent study conducted by Mrabet et al. [23] presented performance analysis of an OCDMA system for LR-PON systems, considering MAI, receiver noise, and single-mode fiber (SMF) channel effects. A mathematical model representing 2D optical code parameters for different receiver structures using Matlab simulations was developed including effects of channel imperfections, such as attenuation losses and chromatic dispersion. Probability of error was investigated for back-to-back (B2B) single mode fiber (SMF) with conventional correlation receiver (CCR) and SMF channel with successive interference cancelation (SIC) receiver. Performance improvement, in terms of number of simultaneous users with Q factor (6) at fiber distances 190 and 120 km, was achieved without amplification.

The rest of this manuscript is arranged as follows. Section 2 describes the construction and properties of the 2D wavelength/time permutation vectors’ codes. Section 3 presents the architecture of the transmitter and receiver of the proposed code. Section 4 analyzes the system performance. The numerical and simulation results are presented in Section 5 and Section 6, respectively. Finally, the conclusions are drawn in Section 7.

## 2. The 2D Wavelength-Hopping/Time-Spreading System

Utilizing a wavelength-hopping/time-spreading (WHTS) as encoding technique for 2D representation provides numerous advantages over other incoherent schemes such as cardinality and code interference mitigation. In this approach, the codes were spread in both the time and wavelength domains simultaneously.

**Figure 1:**
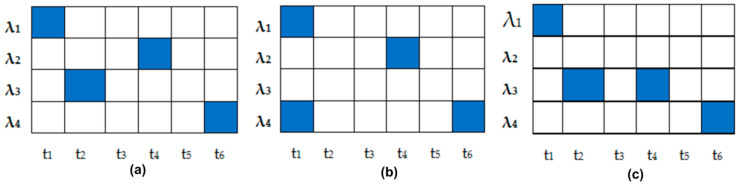
Classification of 2D W-T PV codes (**a**) (λ_1_ λ_3_ 0 λ_2_ 0 λ_4_) (**b**) ({λ_1_, λ_4_} 0 0 λ_2_ 0 λ_4_) (**c**) (λ_1_ 0 λ_3_ 0 λ_3_ 0 λ_2_). * λ = Wavelength.

Three different cases were demonstrated to represent the wavelength-time slots matrices in Figure 1. Firstly symmetric case (SC) is shown in Figure 1a, in which all assigned wavelengths were utilized and every wavelength appeared only once in each code sequence at different time slots. Secondly, asymmetric case (AS) occurred if at least two different wavelengths were located at the same time slot or if the same wavelength appeared twice or more at different time slots (Figure 1b,c).

## 3. The 2-Dimensional Wavelength-Time (W-T) Permutation Vectors’ (PV) Code Construction and Properties

The new 1D PV code was constructed based on permutation vectors. It is characterized by the code length (*L*), the code weight (*w*), and by zero cross-correlation. *L* depends on the number of users (*K*) and *w*. The relationship of these variables is given as follows:

(1) L=wK.

### 3.1. One-Dimensional Approach

Let ℝ refer to the field of real numbers. The space of all *m*-tuples of real numbers forms an *m*-dimensional vector space over ℝ and represented by ${\mathbb{R}}^{m}$. The dimension of the vector space *U* over the field ℝ can be written as dim ℝ (U) or as [U: ℝ]. An element *U* of ${\mathbb{R}}^{m}$ can be represented as vertical vector:

(2) $$U=\left\{\begin{array}{c} u_{1}\\ u_{2}\\ .\\ .\\ u_{m} \end{array}\right\}.$$

Therefore, the dim ℝ(ℝ^3^) = 3. In general, dim ℝ(ℝ^m^) = m.

Using the standard unit basis vectors, any vector $U=\left(\begin{array}{c} u_{1}\\ u_{2}\\ .\\ .\\ u_{m} \end{array}\right)\in {\mathbb{R}}^{m}$ can be expressed as a linear combination $U=u_{1}e_{1}+u_{2}e_{m}+\ldots +u_{m}e_{m}$, where $e_{1},\;e_{2},\ldots ,\;e_{m}$ are the standard unit basis vectors of ℝ^m^.

Let T:$\;{\mathbb{R}}^{m}\to {\mathbb{R}}^{m}$ be a linear transformation defined such that:

(3) $$T\left(e_{1}\right)=\left(\begin{array}{c} 1\\ 0\\ 0\\ .\\ .\\ 0 \end{array}\right);\;T\left(e_{2}\right)=\left(\begin{array}{c} 0\\ 1\\ 0\\ .\\ .\\ 0 \end{array}\right);\ldots \ldots ,T\left(e_{m}\right)=\left(\begin{array}{c} 0\\ 0\\ 0\\ .\\ .\\ 1 \end{array}\right).$$

The representation matrix is:

(4) $$\left[T\left(e_{1}\right),\;T\left(e_{2}\right),\ldots ,T\left(e_{m}\right)\right]=\left(\left(\begin{array}{c} 1\\ 0\\ 0\\ .\\ .\\ 0 \end{array}\right);\left(\begin{array}{c} 0\\ 1\\ 0\\ .\\ .\\ 0 \end{array}\right);\ldots .;\left(\begin{array}{c} 0\\ 0\\ 0\\ .\\ .\\ 1 \end{array}\right)\right),$$

For example, the vector space ℝ^4^ is represented as:

$$\left[T\left(e_{1}\right),\;T\left(e_{2}\right),T\left(e_{3}\right),T\left(e_{4}\right)\right]=\left(\left(\begin{array}{c} 1\\ 0\\ 0\\ 0 \end{array}\right);\left(\begin{array}{c} 0\\ 1\\ 0\\ 0 \end{array}\right);\left(\begin{array}{c} 0\\ 0\\ 1\\ 0 \end{array}\right);\left(\begin{array}{c} 0\\ 0\\ 0\\ 1 \end{array}\right)\right)$$

A permutation vector is a 1 × *n* or *n* × 1 vector of the integers 1 through *n*. The following permutation matrix and permutation vector are equivalent:

$$Perm=\left[\begin{array}{c} 1\\ 2\\ \begin{array}{c} 3\\ 4 \end{array} \end{array}\right]\Leftrightarrow \left[T\left(e_{1}\right),\;T\left(e_{2}\right),T\left(e_{3}\right),T\left(e_{4}\right)\right]=\left(\left(\begin{array}{c} 1\\ 0\\ 0\\ 0 \end{array}\right);\left(\begin{array}{c} 0\\ 1\\ 0\\ 0 \end{array}\right);\left(\begin{array}{c} 0\\ 0\\ 1\\ 0 \end{array}\right);\left(\begin{array}{c} 0\\ 0\\ 0\\ 1 \end{array}\right)\right)$$

Based on the above definitions and given the number of users *K* and the weight *W*, we can generate all possibilities of 1D-PV by getting all permutations of the vectors with repetition of $\left[T\left(e_{1}\right),\;T\left(e_{2}\right),\ldots ,T\left(e_{k-1}\right)\right]$ each vector *w* times. Hence, the 1D-PV codes consists of K×l matrix functionally depending on the value of the number of users (*K*) and code weight (*w*). These 1D-PV codes were constructed based on the vector space ℝ^k^ and an arbitrary permutation vector (Perm).

(5) $$1D-\mathrm{PV}={\left({\mathbb{R}}^{k}|Perm\right)}_{k\times l},$$

where *Perm* is a permutation vector used to permute the columns of the representation matrix of ℝ^k^, the permuted ℝ^k^ is obtained using Equation (6):

(6) $${\overset{´}{\mathbb{R}}}^{k}={\mathbb{R}}^{k}\left[.,Perm\right].$$

Moreover, the proposed 1D−PV is designed such that:

- Cross-correlation between each row is equal to 0.
- Each column is an element of the vector space ℝ^k^.
- Number of *Perm* possibilities is equal to $PV_{poss}=$ $\frac{\left(\mathrm{WK}\right)!}{\left(w!\left(L-w\right)!\right)}$.

Thus, the basic form of 1D−PV for k = w = 2 while using the above-mentioned properties can be written in six forms as:

$${\left(1D-\mathrm{PV}\right)}_{1}=\left({\mathbb{R}}^{2}|\left(1,1,2,2\right)\right)=\left[T\left(e_{1}\right),\;T\left(e_{1}\right),T\left(e_{2}\right),T\left(e_{2}\right)\right]=\left(\begin{array}{cccc} 1 & 1 & 0 & 0\\ 0 & 0 & 1 & 1 \end{array}\right)$$

$${\left(1D-\mathrm{PV}\right)}_{2}=\left({\mathbb{R}}^{2}|\left(1,2,1,2\right)\right)=\left[T\left(e_{1}\right),\;T\left(e_{2}\right),T\left(e_{1}\right),T\left(e_{2}\right)\right]=\left(\begin{array}{cccc} 1 & 0 & 1 & 0\\ 0 & 1 & 0 & 1 \end{array}\right)$$

$${\left(1D-\mathrm{PV}\right)}_{3}=\left({\mathbb{R}}^{2}|\left(1,2,2,1\right)\right)=\left[T\left(e_{1}\right),\;T\left(e_{2}\right),T\left(e_{2}\right),T\left(e_{1}\right)\right]=\left(\begin{array}{cccc} 1 & 0 & 0 & 1\\ 0 & 1 & 1 & 0 \end{array}\right)$$

$${\left(1D-\mathrm{PV}\right)}_{4}=\left({\mathbb{R}}^{2}|\left(2,2,1,1\right)\right)=\left[T\left(e_{2}\right),\;T\left(e_{2}\right),T\left(e_{1}\right),T\left(e_{1}\right)\right]=\left(\begin{array}{cccc} 0 & 0 & 1 & 1\\ 1 & 1 & 0 & 0 \end{array}\right)$$

$${\left(1D-\mathrm{PV}\right)}_{5}=\left({\mathbb{R}}^{2}|\left(2,1,2,1\right)\right)=\left[T\left(e_{2}\right),\;T\left(e_{1}\right),T\left(e_{2}\right),T\left(e_{1}\right)\right]=\left(\begin{array}{cccc} 0 & 1 & 0 & 1\\ 1 & 0 & 1 & 0 \end{array}\right)$$

$${\left(1D-\mathrm{PV}\right)}_{6}=\left({\mathbb{R}}^{2}|\left(2,1,1,2\right)\right)=\left[T\left(e_{2}\right),\;T\left(e_{1}\right),T\left(e_{1}\right),T\left(e_{2}\right)\right]=\left(\begin{array}{cccc} 0 & 1 & 1 & 0\\ 1 & 0 & 0 & 1 \end{array}\right)$$

An example of PV codes generated with weight w = 2 and K = 4 are given in Table 1. The number of possibilities is 28 sets of different patterns of PV code as a product of permutation operation.

### 3.2. W-T Two-Dimensional PV Approach

The obtained 2D W-T OCDMA codes were generated either by using a mathematical approach or by extension of an existing one-dimensional code. In the proposed work, 2D W-T PV codes were obtained by using one-dimensional PV codes for both wavelength-hopping and time-spreading. The new codes can be constructed as follows.

First, we construct *M* groups of PV codes as follows where *M* is ranging between 1 and $PV_{poss}$.

(7) $$PV=\left\{G_{1},G_{2},G_{3},\ldots \ldots .G_{M}\right\},$$

where $G_{i}$ is 1D-PV code constructed based on the vector space ℝ ^k^ and an arbitrary permutation vector (Permi), so that: 
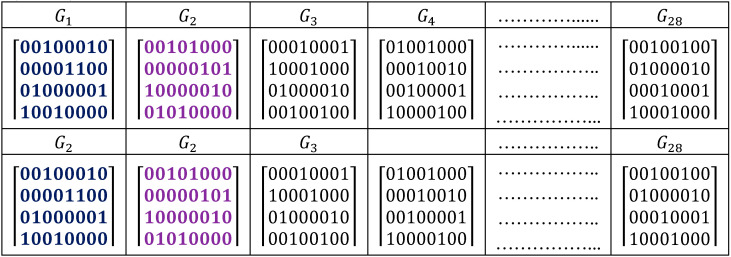

$$G_{i}={\mathbb{R}}^{k1}\left[.,Permi\right]=\left\{C_{i1},C_{i2},C_{i3},\ldots \ldots .C_{ik}\right\},$$

where, $C_{ij}$ is a 1D-PV code where i∈[1, M] and j∈[1, K] and *K* is the number of generated codes in the group. Hence $C_{ij}$ is an arbitrary code in $G_{i}$. The 2D W-T PV codes can be constructed by selecting $G_{i}$ to generate *L* time slots. Each $C_{ij}$ will be used as time-spreading patterns to determine the positions of the wavelengths. For the wavelength-hopping patterns, another 1D-PV code group was used $G_{d},$ where *d* ≠ *i*. Hence, the wavelength-hopping patterns form the wavelength’s index. Between two selected groups (*G_i_* and *G_d_*), the total number of served users = K^2^. Therefore, the 2D W-T PV codes’ generation equation was defined as:

(8) $$A_{d,i}=G_{d}^{T}G_{i}=\left(\begin{array}{ccc} C_{d1}C_{i1} & \cdots & C_{d1}C_{ik}\\ \vdots & \ddots & \vdots \\ C_{dk}C_{i1} & \cdots & C_{dk}C_{ik} \end{array}\right).$$

Each element in $A_{d,i}$ is a square matrix (*L* × *L*), where *L* represents the number of available wavelengths and the number of time slots. The 2D W-T PV codes matrices were obtained by associating the rows with wavelengths and the columns with time slots. For example, choose i = 1 and d = 2 (group 1 and group 2 from Table 1). Then the code matrix $C_{21}C_{11}$ is represented as follows.

C21C11=0000000000000000000000000000001000000000001000000000000000000000wavelength’s×time

**Figure 2:**
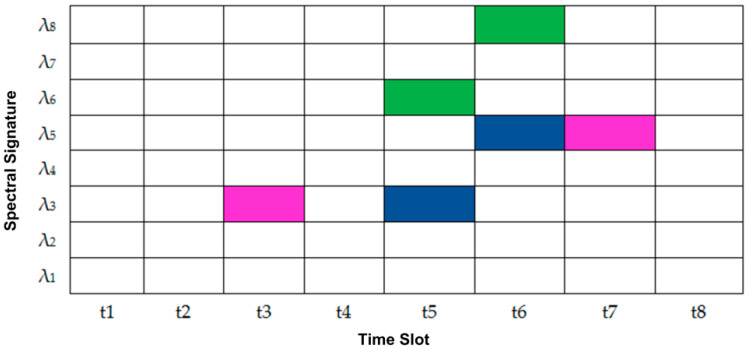
Code’s illustration $C_{21}C_{11}$, $C_{21}C_{12},$ and $C_{22}C_{12}$.

**Figure 3:**
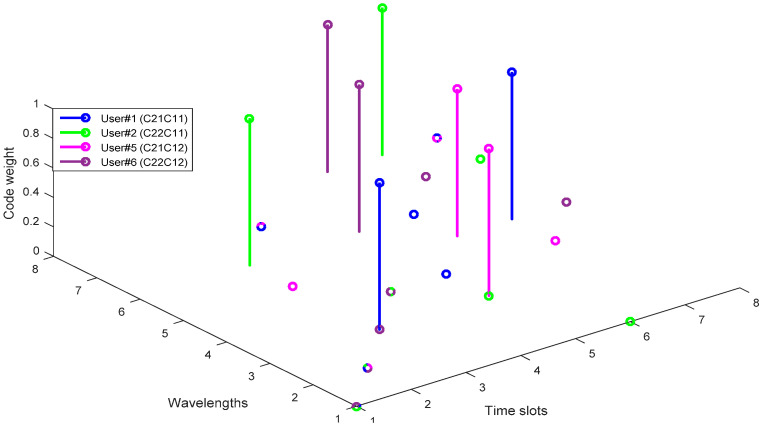
A 3-dimensional (3D) graphic representation of 2D W-T PV code sequences.

As shown in Figure 2 and Figure 3, the 2D W-T PV generated codes were differentiated with either the wavelength’s hopping or the time spreading.

For example, from Figure 2, it is clear that the codes $C_{21}C_{11}$ (pink colored) and $C_{21}C_{12}$ (blue colored) shared the same spectral signature (λ_3_, λ_5_) but transmitted them at different time slots (t3, t7) and (t5,t6), respectively. Furthermore, when two codes used the same time slots, like $C_{21}C_{12}$ and $C_{22}C_{12}\;$(green colored), they sent different wavelengths, (λ_3_, λ_5_) for $C_{21}C_{12}$ and (λ_6_, λ_8_) for $C_{22}C_{12}$, thus maintaining a zero cross-correlation.

Figure 3 shows a 3-dimensional (3D) graphic representation of two 2D W-T PV code sequences, which represent either time spreading or wavelength hopping. It is clear that user#5 and user#6 shared the same spectral signature with spreading at different time slots.

### 3.3. Properties of 2D W-T Permutation Vectors (PV) Codes

In order to define the cross-correlation of a 2D W-T PV code, four matrices are defined as Equations (10)–(13).

(9) $$A_{g,h}{}^{\left(0\right)}=G_{h}{}^{T\;\;}G_{g}$$

(10) $$A_{g,h}{}^{\left(1\right)}={\bar{G_{h}}}^{T}G_{g}$$

(11) $$A_{g,h}{}^{\left(2\right)}=G_{h}{}^{T}\bar{G_{g}}$$

(12) $$A_{g,h}{}^{\left(3\right)}={\bar{G_{h}}}^{T}\bar{G_{g}}.$$

The cross-correlation between any arbitrary code, $A_{d,i}$, and characteristic code, $A_{g,h},$ can be defined through Equation (14).

(13) $$R_{g,h}^{p}\left(d,i\right)=\overset{L-1}{\underset{f=0}{\sum }}\overset{L-1}{\underset{j=0}{\sum }}a_{\left(d,i\right)}\left(f,j\right)a_{\left(g,h\right)}^{\left(p\right)}\left(f,j\right),$$

where $a_{\left(g,h\right)}^{\left(p\right)}$ and $a_{\left(d,i\right)}$ represent the elements of $A_{g,h}{}^{\left(p\right)}$ and $A_{d,i}$ 2D W-T PV matrix, respectively, and p ∈[0,…,3]. In accordance with Equation (11), the cross-correlation between any two codes $A_{g,h}$ and $A_{d,i}$ can be calculated as shown in Table 2.

As the cross-correlation along $G_{i}$ and $G_{d}^{T}$ code sequences equals zero, so $R_{g,h}^{\left(1,3\right)}\left(d,i\right)$ has no use for the new correlation function, which makes the multiple user interference (MUI) elimination process easier. Hence, the expression of a new cross-correlation function from the previous table is given as follows:

(14) $$R_{g,h}^{p}\left(d,i\right)=\{\begin{array}{c} w,\;for\;d=g,\;i=h\\ 0\;otherwise\;\;\;\;\;\;\;\;\;\;\;\; \end{array}.$$

## 4. 2-Dimensional PV System Description

### 4.1. Design of 2D-PV System

The basic 2D-PV-based OCDMA system comprises numerous W-T encoders/decoders at the transmitter and receiver parts, respectively. Each encoder/decoder structure is made up of FBG-based delay lines, each with a certain bit period. FBGs as delay lines are preferred due to low circuit complexity and reduced delay time when compared to first generation 2D TW implementations (tunable filters and delay lines) [24]. Due to recent advancement in thin film technology, thin film filters (TFFs) are other alternative implementation approaches [25].

### 4.2. Design of 2D-PV Transmitter Part

**Figure 4:**
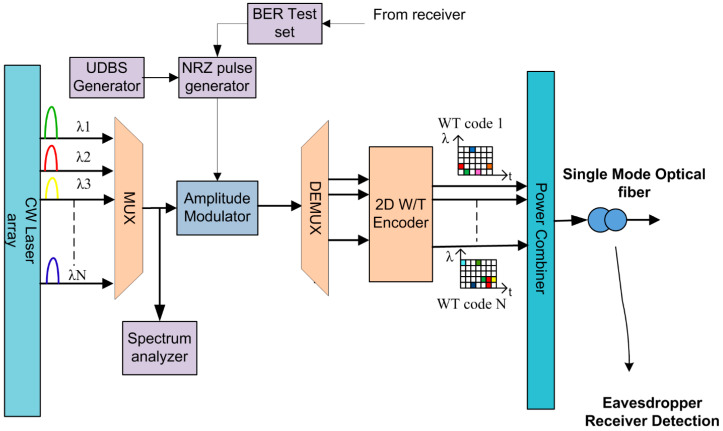
Transmitter structure for 2D W-T PV optical code division multiple access system.

The conventional transmitter structure for a W-T OCDMA system is shown in Figure 4. Initially, the transmitter module consists of a multi-wavelength continuous wave (CW) laser array as an optical source. For each user, a group of wavelengths is produced by the CW laser. These short pulse trains are set to a width τ ((τ = *t_b_*/S), which represents code length in time spread), where *t_b_*/S stands for bit time period per second. The pulse train repetition rate is set equal to the system bit rate (1/*t_b_*). The bit stream is modulated by the input data using an amplitude modulator. In the on-off keying process, the modulator produces an optical pulse when transmitted data bit is one, or else it produces zero output.

Modulated signal/data pulses are demultiplexed using a demultiplexer. Pulses corresponding to data bit one are sent to a 2D W-T-based optical encoder to perform encoding operation. The W-T encoder circuit is made up of time delay circuits and Bessel optical filters. In 2D TW codes, chosen pulses are encoded by different or similar spectral components according to the PV code algorithm (Table 1). Each optical pulse corresponding to transmitted bits is divided into w optical pulses with time delay (*t*) between each one of them. Finally, an encoded data stream (wavelength time-spreading patterns) is transmitted to a single-mode optical fiber through a power combiner.

### 4.3. Design of 2D-PV Receiver Part

**Figure 5:**
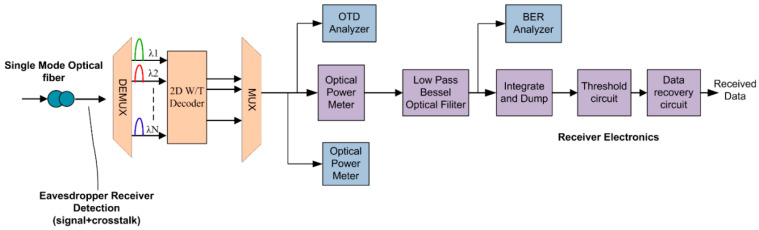
Receiver structure for 2D-PV OCDMA system.

The receiver structure for a conventional 2D W-T OCDMA system is shown in Figure 5. The fiber output data is demultiplexed and sent to a 2D PV decoder circuit, which consists of low-pass Bessel filters and inverse time delay circuitry. Direct detection with a single-photo diode detector or balance detection technique can be employed for signal detection with a certain threshold level set to achieve the desired output. Finally, at the receiver, both input and output data are compared by BER test set and, therefore, BER analysis is carried out by BER test set. A Spectrum analyzer and optical time domain analyzer (OTDA) are used to analyze the changes in signal power and to measure spectrum of various signals with time at different points of the system.

## 5. Proposed 2D-PV W-T-Based OCDMA-PON

**Figure 6:**
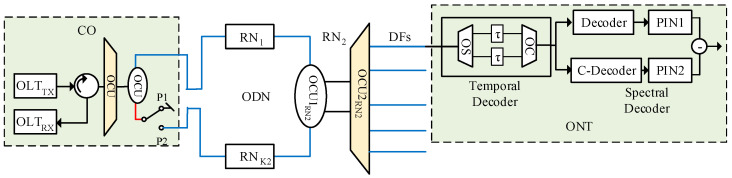
Proposed 2D PV-based OCDMA-Passive optical network architecture.

Figure 6 shows the proposed architecture that incorporates a maximum of $K_{1}\times K_{2}$ transmitters, $N_{2}$ remote nodes, and $K_{1}\times K_{2}$ receiver modules. Here, $K_{1}$ and $K_{2}$ represent the number of spectral and temporal code word, whereas $N_{2}$ is the temporal code length. Each subscriber is assigned with a part of the transmitter and receiver module corresponding to a 2D vector permutation code word $A_{g,h}$. Furthermore, a hybrid ring and tree-based topology is adapted at the feeder and distribution level to facilitate deployment of the proposed architecture as a low-cost PON. Optical source power is set to 0 decibel-milliwatts (dBm) with data bit rate 1 Giga bit per second (*t_b_* = 1 ns).

**Figure 7:**
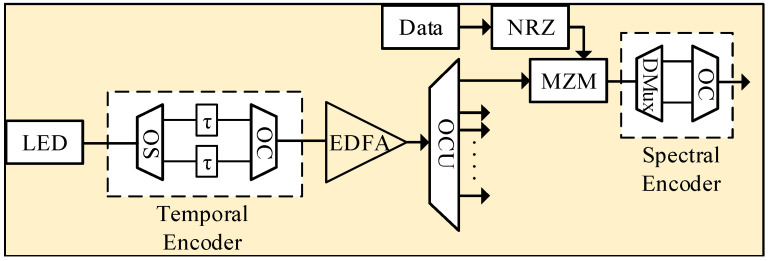
Transmitter module.

Figure 7 illustrates construction of the transmitter module in correspondence with the proposed 2D VP code word, $A_{g,h}$. For illustration purpose, an example of a single user $U_{0,0}$ was considered, as shown in Table 3 with a $X^{th}$ and $Y^{th}$ code words of [0 0 1 0 0 0 1 0] and [0 0 1 0 1 0 0 0], respectively. In addition, Table 3 shows the allocation of respective wavelengths $\left(\lambda_{s}\right)$ and time delays $\left(\tau_{s}\right)$ for corresponding chips in the spectral and temporal code sequences, respectively. For example, for user $U_{0,0}$, the spectral encoding was achieved by allowing the wavelengths centered at $\lambda_{3}$ and $\lambda_{7}$, whereas the temporal encoding was achieved by employing $\tau_{3}$ and $\tau_{5}$ delay instants. Furthermore, for the user $U_{0,0}$ under consideration, the transmitter modules incorporate a combination of broadband light sources (BBS), temporal and spectral encoders, erbium-doped fiber amplifier (EDFA), modulation, and switching arrangement.

Initially the broadband spectrum generated by the BBS, called light emitting diode (LED), is delivered to the temporal encoding arrangement, which contains a $1:w_{2}$ optical splitter $\left(OS_{TX}\right)$. It can be observed from Table 3 that when h=0 in $A_{g,h}$, all the $X^{th}$ code words were delayed by the same instant. Therefore, employing a single time delay unit, in comparison with the conventional technique, can significantly reduce the complexity and cost of the system. $OS_{TX}$ splits the incoming signal into $w_{2}$ equal portions, which are then applied to the time delay units (TUs), as shown in Figure 6. The $TU_{TX}$ across each line for user $U_{0,0}$ was proportional to the location of one chip of the time-spreading code word $Y_{h}$, respectively, as shown in Table 3. For example, in the placement of chips in the time-spreading codeword $Y_{1}^{T}=\left\{3,5\right\},$ suppose unit delay equals to *τ*, then the delay time of the $TU_{TX}$ for $Y_{1}$ is (3τ, 5τ), respectively. End faces of both the $TU_{TX}$ were combined via $w_{2}:1$ optical combiner ($OC_{TX}$) to complete the time-spreading encoding for the code words $A_{g,0}$, respectively.

Further, the output of the time-spreading encoder was delivered to an EDFA and $1:N_{K_{1}}OS_{TX}$, where $N_{K_{1}}$ corresponds to the number of users with reference to the spectral code words. EDFA was employed prior to the splitting arrangement in order to compensate the splitter losses and maintain the integrity of the time-spread optical pulses. For user $U_{0,0}$, the first port of the $1:N_{K_{1}}OS_{TX}$ was delivered to the modulating arrangement where the Mach-Zhender modulator (MZM) modulates the incoming data bits with the optical signal by using an ON-OFF keying (OOK) format.

The modulated signal was delivered to the second stage of the encoding operation, namely, the spectral encoder, which was formed by the combination of $1:w_{1}$ OS and $w_{1}:1$ multiplexer (MUX). Each leg of the $MUX_{w_{1}:1}$ contained a bandpass filter that was tuned to allow the required spectrum with correspondence to the chip placement in $X^{th}$ code sequence, as shown in Table 3. Consequently, for user $U_{0,0}$, the spectral encoder allowed the spectrum located at $\lambda_{3}$ and $\lambda_{7}$, respectively. Consequently, the 2D temporal and spectral encoding operation for the coder word $A_{0,0}$ of user $U_{0,0}$ was achieved at the transmitter module.

Then, the end face of the spectral encoder was connected to a switching arrangement (SWA) via $N_{K_{1}}N_{K_{2}}:1$ optical coupler $\left(OCU_{TX}\right)$. The SWA was deployed to exploit the built-in redundancy of the ring topology and ensure desired connection availability at the feeder level. The SWA, as shown in Figure 6, contained a combination of $1:2\;OCU_{SWA}$ and an optical switch, respectively, with port 1 as the input and ports 2 and 3 as the output ports, respectively. The $1:2\;OCU_{SWA}$ received the encoded spectrum from $N_{K_{1}}N_{K_{2}}:1$ $OCU_{TX}$ via input port 1. The encoded spectrum was equally forwarded towards the output ports 2 and 3, respectively. Port 2 of the $1:2\;OCU_{SWA}$ extended towards the optical distribution network (ODN) that further connected with multiple remote nodes, whereas port 3 was fed to an optical switch $1:2\;SW_{SWA,}$ as shown in Figure 6.

Under normal working conditions, port 2 of the $SW_{SWA}$ is connected to an optical null and all traffic propagates in a clockwise direction towards the ODN, as shown in Figure 6 using red fiber. However, in a case of failure or cuts in the feeder fiber, Optical line terminator (OLT) unit directs the $SW_{SWA}$ to change its position from port 2 to port 3, which provides a clockwise and counterclockwise flow traffic until the point of failure. Consequently, the proposed architecture can provide extended connection availability along with high spectral efficiency in comparison with the existing architecture.

ODN essentially contains a combination of ring-based feeder fiber $\left(FF_{R}\right)$, remote nodes $\left(RN_{K_{2}}\right)$, distribution fibers $\left(DF_{N}\right)$, and optical network terminals (ONTs). One end of the $FF_{R}$ relates to port 2 of the $OCU_{SWA}$ in order to carry the traffic towards the ODN in a clockwise direction under a normal mode of operation, whereas, the other end terminated at port 3 of the $SW_{SWA}$, as shown in Figure 6. $FF_{R}$ also contains multiple $RN_{K_{2}}$ to carry the traffic from feeder level to the respective ONTs at the distribution level.

Each $RN_{K_{2}}$ consisted of two OCUs with the split ratio of $1:2\;\left(OCU1_{RN_{1}}\right)$ and $1:K_{1}\left(OCU2_{RN_{1}}\right),$ respectively. For user $U_{0,0}$, $RN_{1}$ was considered, as shown in Figure 6. The $OCU1_{RN_{1}}$ received the $FF_{R}$ through its input port 1 and split the incoming signal into two equal portions. One portion (via port 2 of the $OCU1_{RN_{1}}$) of the incoming signal was sent towards the $OCU2_{RN_{1}}$, whereas another portion (via port 3 of the $OCU1_{RN_{1}}$) was forwarded towards the $RN_{2}\;($Figure 6).

The spectral/temporal multiplexed signal was received from port 2 of the $OCU1_{RN_{1}}$ via the input port of the $OPCU2_{RN_{1}}$. The second optical coupler at $RN_{N}$ had a split of $1:K_{1}$, which was used to carry the encoded spectrum towards the concerned ONTs via their respective DFs.

Port 3 of the $OCU1_{RN_{1}}$ further extended the ring-based fiber and was connected to the $RN_{2}$, which consisted of the similar arrangement as discussed earlier. Since the number of $OCU1_{RN_{s}}$ used throughout the network to support the given number of users was dependent of the value of $Y^{th}$ code sequences, it was of prime importance to keep the number of codes as few as possible to address the power constraints of the ring-based topology.

The ONT module for the proposed 2D architecture employed a time-based decoding arrangement, which was designed in accordance with the temporal encoder. It split the incoming signal into two equal portions while using a 1:2 $OS_{TD}$. Each leg of the $OS_{TD}$ was connected to a TU that was calculated as (S−1−j), where j is the chip position in the $Y_{0}^{T}$ code sequence for user $U_{0,0}$. Outputs from the TUs were combined with the help of a 1:2 $OC_{TD}$ and forwarded for spectral decoding.

The spectral decoder essentially contained a balanced decoding arrangement commonly referred to as complementary subtraction detection schemes (CSD). CSD for the proposed architecture consisted of a 1:2 $OS_{ONT}$ that split the time decoded-signal into two equal portions. One portion was fed to a filter arrangement in the upper leg of the CSD decoder, whereas another portion was fed to another filter arrangement at the lower leg of the decoder, as shown in Figure 6. Filters in the upper leg of the CSD decoder contained a combination of fiber Bragg-grating (FBG) filters that were centered in accordance with the $X_{1}$ code spectral signature.

The lower leg of the CSD decoder employed a combination of FBG filters configured to complement to the 1s in the $X_{1}$ code sequence. In other words, the lower leg of the CSD decoder for user $U_{0,0}$ extracted the spectrum that was in complement with the $X_{1}$ code sequence. Then, resulting signals from both legs of the spectral decoder related to respective PIN photodiodes in order to convert the signal from optical to electrical domain for necessary processing. The output from the PIN photodiodes was further connected to the subtractor arrangement that subtracted the output from both legs of the CSD decoder to recover the intended spectrum with maximum power units for the intended subscribers and 0 power units for interfering users.

## 6. Proof of Concept

This section analyzes the performance of the proposed setup through system implementation in an optical networks simulation software called Optisystem. The proposed system architecture presented in Figure 6 and Figure 7 was utilized to implement the simulation model for eight subscribers accessing the medium simultaneously. To deploy eight subscribers across the network, four spectral coding schemes and two temporal schemes were utilized. Consequently, the simulation model was implemented with $K_{1}=4,{\;K}_{2}=2$ at g = 0, 1, 2, 3 and h = 0, 1.

The OLT module was implemented in accordance with Figure 7, which shows a transmitter module configured at $g=0,\;1,\;2,\;\ldots ,{\;K}_{1}-1$ and h = 0. As mentioned earlier, for h=0 in $A_{g,h}$, all the spectral code sequences could be delayed by the same instant. Therefore, two LEDs followed by two temporal decoders were employed to configure encoding arrangements for eight subscribers accessing the medium simultaneously. Now, for h = 0, four subscribers were configured with a single broad-spectrum LED that related to time delay units through a 1:2 optical splitter having 0 dB loss. Both legs of the optical splitter were fed into time delay units that were configured in accordance with the position of 1s in the $Y_{0}^{T}$ coding scheme. For instance, the TU connected with the top leg of the splitter was delayed by an amount of $\frac{3}{\mathrm{Bit}\;\mathrm{rate}*8}$, whereas the bottom TU was configured with the delay of $\frac{5}{\mathrm{Bit}\;\mathrm{rate}*8}$, respectively. Similarly for h = 1, the TUs were configured with the delays of $\frac{6}{\mathrm{Bit}\;\mathrm{rate}*8}$ and $\frac{8}{\mathrm{Bit}\;\mathrm{rate}*8}$ in accordance with the position of 1s at the $Y_{1}^{T}$ coding scheme.

The end face of the TUs were connected to a 2:1 optical combiner with 0 dB loss, followed by an EDFA module. Specification of the system components used during simulation analysis are given as Table 4. The amplified time-delayed signal from EDFA module related to a 1:4 optical coupler, in accordance with $K_{1}=4$. Each output leg of the coupler was fed into an individual MZM in order to modulate the signal with the user’s information. After modulation, the process of spectral encoding was initiated by employing a combination of 1:2 optical splitter and MUX arrangement. MUX arrangement for each subscriber was configured to reflect the frequency bins in accordance with position of 1s in the $X_{g}$ code sequence. Then, for user $U_{0,0}$, the spectral encoder allowed the spectrum located at $\lambda_{3}{\;\mathrm{and}\;\lambda }_{7},$ respectively. Similarly, for user $U_{0,1}$, a spectral encoding arrangement with same filter configuration was used to allow the spectrum located at $\lambda_{3}{\;\mathrm{and}\;\lambda }_{7},$ respectively. Consequently, the 2D temporal and spectral encoding operation for the coder word $A_{0,0}$ till $A_{3,1}$ was achieved at the transmitter module.

Outputs from the spectral encoders were connected to an 8:1 optical coupler followed by 1:2 coupler having 0 dB loss and were configured in accordance with Figure 6. Moreover, two remote nodes were employed in the simulation model, in accordance with $K_{2}=2$. The ONT module for the simulation arrangement was configured accordingly to recover and detect the intended spectrum, in accordance with the proposed coding scheme. Each ONT module started with a 1:2 optical splitter to initiate the process of temporal decoding. Each leg of the splitter was fed to a TU with delays calculated through (S−1−j). The end face of each TU related to a 2:1 optical combiner to conclude the process of temporal decoding.

This process was followed by spectral decoding that was implemented with the help of balanced detectors. Balanced detectors split the incoming signal into two parts. One part was applied to an arrangement of FBG filters that were configured in accordance with spectral signature of the encoder. For example, for user $U_{0,0},$ FBG filters at the top leg were configured to recover $\lambda_{3}{\;\mathrm{and}\;\lambda }_{7}$, whereas the FBG filter employed at the bottom leg was used to recover the complement of the intended signature. The output of both filter arrangements was passed through PIN photodiodes and subtracted to recover the intended spectrum with maximum auto- and minimum cross-correlation.

**Figure 8:**
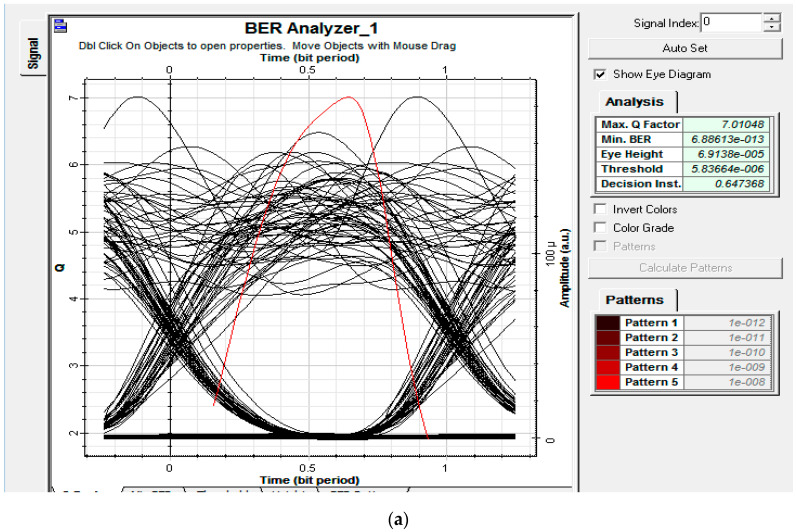

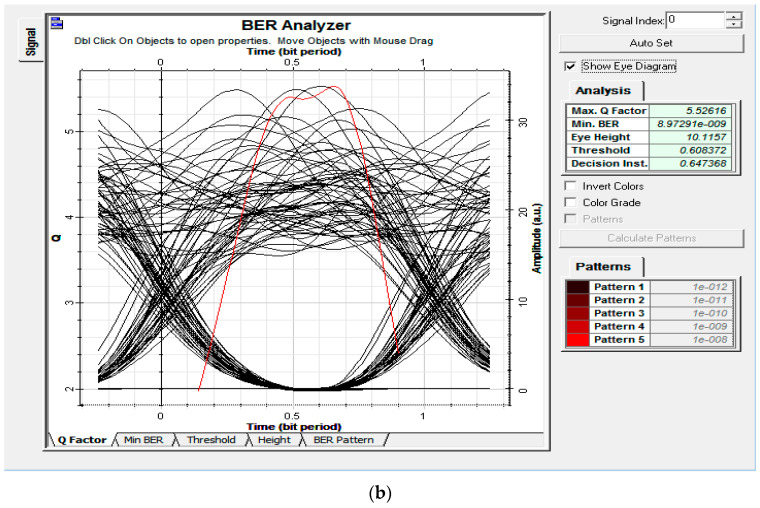
Eye diagrams for downlink traffic at (**a**) 1 Gbps and (**b**) 2 Gbps.

Then, for the initial analysis, the performance of the proposed system was analyzed in terms of data rate versus bit error rate. Moreover, with an EDFA of 9 dB gain, power at the ONTs of the first remote node $RN_{1}$ was set at 0 dBm, respectively. Figure 8 shows the eye diagrams and BER of the proposed setup at 1 Gbps and 2 Gbps of data for randomly selected nodes of $ONT_{RN_{1}}$. It was observed that the proposed setup was well able to support high data rates of up to 2 Gbps. Moreover, analysis of the BER and eye diagrams validated the implementation setup such that BER increased with an increase in the amount of data transmitted between the OLT and ONTs’ modules.

This can be attributed to the fact that pulse width decreased with an increase in the data bits, which made the system more vulnerable to distortion across the medium. Consequently, a higher data rate will yield a higher BER. However, analysis showed that for an agreeable BER of $10^{-9}$ the proposed setup was able to provide the desired performance up to 2 Gbps of data, owing to the efficient cross-correlation properties at the $X^{th}$ code sequence and proficient design of the proposed setup that maintained a nominal signal power to yield a desired signal-to-noise ratio and, hence, BER.

**Figure 9:**
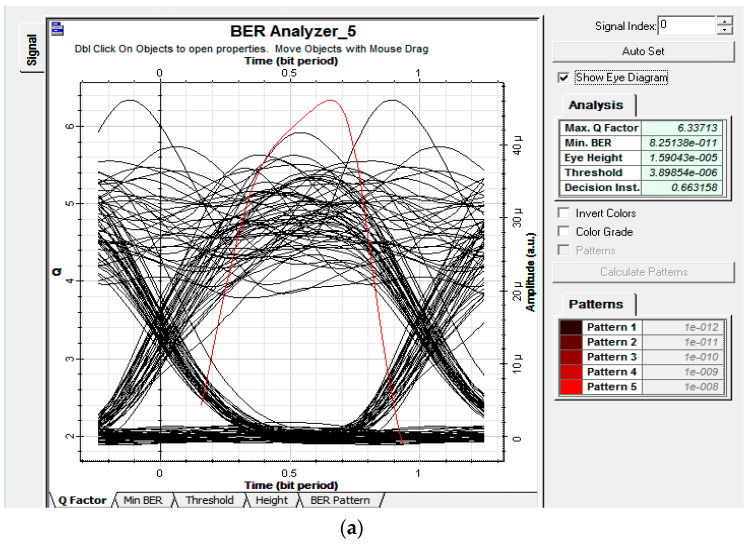

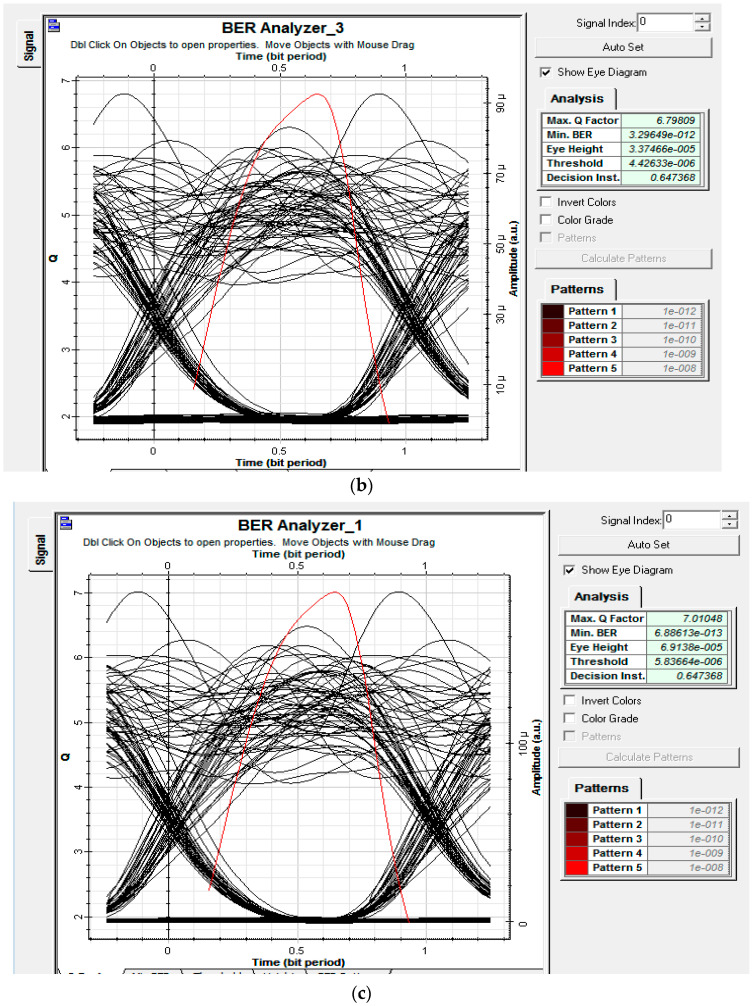
Eye diagrams for downlink traffic at (**a**) −6 dBm, (**b**) −3 dBm, and (**c**) 0 dBm.

To further analyze the impact of the power budget on the overall performance of the setup, analysis was made at 1 Gbps of data by randomly selecting nodes at each remote node from $RN_{1}-RN_{3}$. Moreover, through the application of nine EDFA at the transmitter module, 0 dBm, −3 dBm, and −6 dBm power was observed at the $ONT_{RN_{1}}$, $ONT_{RN_{2}}$, and $ONT_{RN_{2}}$, respectively. Eye diagrams and BER analysis in Figure 9 show that the proposed setup was capable enough to handle the power drops at each remote node and provide desired results for an acceptable BER of $10^{-9}$. Results’ analysis also validated the proposed model, which showed a relevant decrease in the BER along with a decrease in the power each ONT of alternated remote nodes.

In order to analyze the impact of the switching arrangement and observe the capability of the proposed setup to handle traffic in both clockwise and counterclockwise directions, the proposed model was simulated with an optical switch after the transmitter module. Moreover, for fair analysis, the number of simultaneous users accessing the medium was reduced to $K_{1}=4,\;K_{2}=2$ with $N_{2}=8$. Analysis was performed at 1 Gbps of data and port 2 of the switch was initially engaged to observe the flow of traffic in a clockwise direction. In the second phase of the simulation, port 3 was engaged to analyze the flow of traffic in a counterclockwise direction to mimic a state of failure.

**Figure 10:**
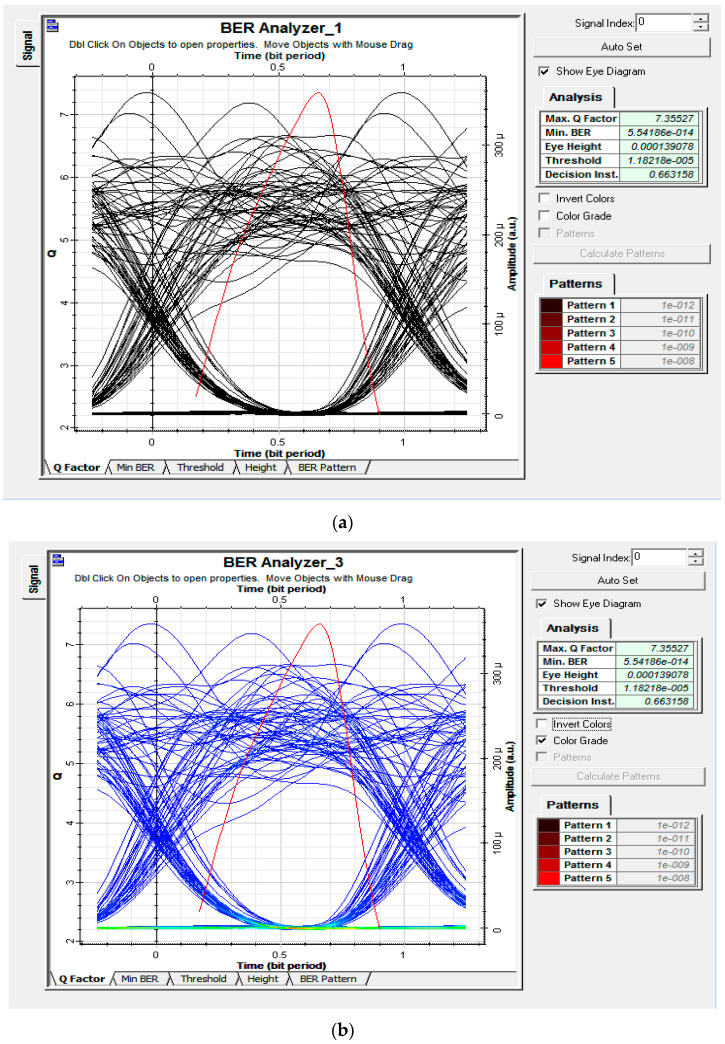
Eye diagrams for downlink traffic at (**a**) clockwise and (**b**) counterclockwise directions.

Figure 10 shows the eye diagrams and BER analysis for the flow of traffic in both clockwise and counterclockwise directions. It is evident that the proposed setup was able to support the flow of traffic in both directions with minuscule effect on the overall performance. It can be observed that for 1 Gbps of data almost the same BER was obtained at the randomly selected ONT of $RN_{1}$ (clockwise flow of traffic) and $RN_{2}$ (counterclockwise flow of traffic). Hence, the proposed VP-based 2D OCDMA system was not only able to provide high capacity in terms of data, reach, and the number of users, but was also capable of providing relative support against feeder fiber failure, which is novel, as compared to the existing 2D OCDMA architecture.

**Figure 11:**
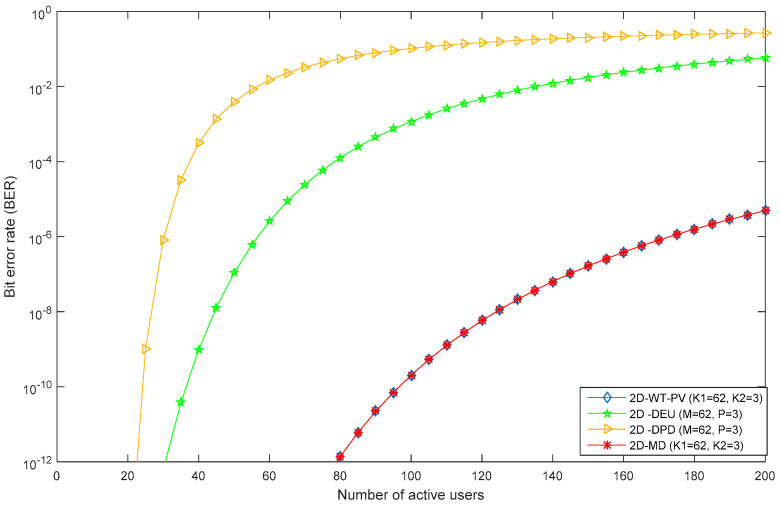
Bit error rate vs. number of active users for 2D PV (K1 = 63, K2 = 3), 2D diagonal eigen value unity code (M = 63, P = 3), 2D diluted perfect difference code (M = 63, P = 3), and 2D Multi Diagonal code (K1 = 62, K2 = 3) with 0 dBm effective transmitted power at data rate 622 Mbit/sec.

The 2D PV code performance, in terms of BER and number of supportable clients, is shown in Figure 11. As the number of users increased the quality of the received signal deteriorated due to the occurrence of MAI among them. Thus, system signal to noise ratio (SNR) and BER degraded. Moreover, BER values of 2D PV code (K1 = 62, K2 = 3; where K1 and K2 refers to number of subscribers in spectral and spatial domain) were compared with existing techniques: Diluted perfect difference code (M = 63, P = 3) and diagonal eigenvalue unity code (M = 63, P = 3; M and P represents number of subscribers in spectral and spatial domain). It was observed that supported clients were 20, 40, and 110 for 2D diluted perfect difference (DPD), 2D diagonal eigen value (DEU), and 2D PV code, respectively, at the minimum acceptable BER value of 10^−9^. In particular, multi diagonal (MD) code (M = 63, P = 3) is also plotted and results indicate an overlap with our proposed code due to their similar properties.

## 7. Conclusions

A new 2D PV coding scheme was proposed based on a WHTS coding approach and possesses several advantages over the existing families, such as simplicity of code construction, no limitation of the selection of any positive integer weight, and high degree of cardinality with increased flexibility. The proposed code can be employed for both synchronous and asynchronous incoherent OCDMA environment. Results indicated that 2D W-T PV code had improved system performance due to reduced error probability with MAI and phase induced intensity noise (PIIN) effects suppression and easier code implementation capabilities. For future implementations, simple direct detection method can be employed in the receiver design, which is cost effective and less complex due to the utilization of single-photo diode and low-pass filter circuit. The numerical results indicated that the 2D PV code provided better performance in terms of system parameters such as transmitted power at the source, cardinality, BER, and data rate. The simulation results demonstrated successful implementation of the 2D PV-based OCDMA scheme and make it applicable for all optical networks in practice by maintaining optimal BER (10^−9^) required for error-free transmission at minimum data rate.

## Figures and Tables

**Table 1:**
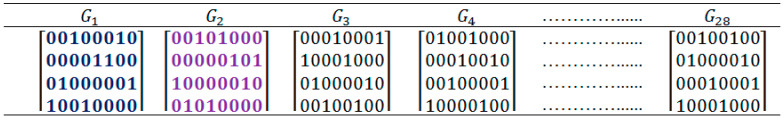
1-dimensional-PV codes generated with code weight (w) = 2 and number of generated codes (K) = 4.

**Table 2:** Cross-correlation values of 2D W-T PV codes.

	$R_{g,h}^{0}\left(d,i\right)$	$R_{g,h}^{1}\left(d,i\right)$	$R_{g,h}^{2}\left(d,i\right)$	$R_{g,h}^{3}\left(d,i\right)$
d=g,i=h	***w***	0	0	0
d=g,i ≠h	0	***w***	0	0
d ≠ g,i=h	0	0	***w***	0
d ≠ g,i ≠h	0	0	0	***w***

**Table 3:** Allocation of respective wavelengths $\left(\lambda_{s}\right)$ and time delays $\left(\tau_{s}\right)$.

$A_{g,h}$		$\lambda_{1}$	$\lambda_{2}$	$\lambda_{3}$	$\lambda_{4}$	$\lambda_{5}$	$\lambda_{6}$	$\lambda_{7}$	$\lambda_{8}$	$\lambda_{9}$	$\lambda_{10}$	$\lambda_{11}$	$\lambda_{12}$	$\lambda_{13}$	$\lambda_{14}$	$\lambda_{15}$	$\lambda_{16}$
		0	0	1	0	0	0	1	0	0	0	0	0	1	1	0	0
$\tau_{1}$	0	0	0	0	0	0	0	0	0	0	0	0	0	0	0	0	0
$\tau_{2}$	0	0	0	0	0	0	0	0	0	0	0	0	0	0	0	0	0
$\tau_{3}$	1	0	0	1	0	0	0	1	0	0	0	0	0	1	1	0	0
$\tau_{4}$	0	0	0	0	0	0	0	0	0	0	0	0	0	0	0	0	0
$\tau_{5}$	1	0	0	1	0	0	0	1	0	0	0	0	0	1	1	0	0
$\tau_{6}$	0	0	0	0	0	0	0	0	0	0	0	0	0	0	0	0	0
$\tau_{7}$	0	0	0	0	0	0	0	0	0	0	0	0	0	0	0	0	0
$\tau_{8}$	0	0	0	0	0	0	0	0	0	0	0	0	0	0	0	0	0
$\tau_{1}$	0	0	0	0	0	0	0	0	0	0	0	0	0	0	0	0	0
$\tau_{2}$	0	0	0	0	0	0	0	0	0	0	0	0	0	0	0	0	0
$\tau_{3}$	0	0	0	0	0	0	0	0	0	0	0	0	0	0	0	0	0
$\tau_{4}$	0	0	0	0	0	0	0	0	0	0	0	0	0	0	0	0	0
$\tau_{5}$	0	0	0	0	0	0	0	0	0	0	0	0	0	0	0	0	0
$\tau_{6}$	1	0	0	1	0	0	0	1	0	0	0	0	0	1	1	0	0
$\tau_{7}$	0	0	0	0	0	0	0	0	0	0	0	0	0	0	0	0	0
$\tau_{8}$	1	0	0	1	0	0	0	1	0	0	0	0	0	1	1	0	0

**Table 4:** Parameters used in simulation analysis.

System Component	Value
LED spectrum	30 nm
LED frequency	1490 nm
EDFA Gain	9 dB
Encoder bandwidth	0.4 nm
MZM extinction ratio	30 dB
SMF attenuation	0.25 dB/km
SMF dispersion	18 ps/nm/km
FF ring length	20 Km
DF length	5 Km
Decoder bandwidth	0.4 nm
PIN responsitivity	0.75 A/W
PIN dark current	10 nA
PIN thermal noise	1e^−22^ W/Hz
